# Macrophage death in atherosclerosis: potential role in calcification

**DOI:** 10.3389/fimmu.2023.1215612

**Published:** 2023-07-04

**Authors:** Jaap G. Neels, Claire Gollentz, Giulia Chinetti

**Affiliations:** ^1^ Université Côte d’Azur, Institut national de la santé et de la recherche médicale (INSERM), Centre Méditerranéen de Médecine Moléculaire (C3M), Nice, France; ^2^ Université Côte d’Azur, Centre Hospitalier Universitaire (CHU), Institut national de la santé et de la recherche médicale (NSERM), Centre Méditerranéen de Médecine Moléculaire (C3M), Nice, France

**Keywords:** macrophages, cell death, calcification, atherosclerosis, plaque stability, cardiovascular disease

## Abstract

Cell death is an important aspect of atherosclerotic plaque development. Insufficient efferocytosis of death cells by phagocytic macrophages leads to the buildup of a necrotic core that impacts stability of the plaque. Furthermore, in the presence of calcium and phosphate, apoptotic bodies resulting from death cells can act as nucleation sites for the formation of calcium phosphate crystals, mostly in the form of hydroxyapatite, which leads to calcification of the atherosclerotic plaque, further impacting plaque stability. Excessive uptake of cholesterol-loaded oxidized LDL particles by macrophages present in atherosclerotic plaques leads to foam cell formation, which not only reduces their efferocytosis capacity, but also can induce apoptosis in these cells. The resulting apoptotic bodies can contribute to calcification of the atherosclerotic plaque. Moreover, other forms of macrophage cell death, such as pyroptosis, necroptosis, parthanatos, and ferroptosis can also contribute by similar mechanisms to plaque calcification. This review focuses on macrophage death in atherosclerosis, and its potential role in calcification. Reducing macrophage cell death and/or increasing their efferocytosis capacity could be a novel therapeutic strategy to reduce the formation of a necrotic core and calcification and thereby improving atherosclerotic plaque stability.

## 1 Introduction

Atherosclerosis and its clinical complications are still a leading cause for morbidity and mortality worldwide. Although significant improvements have been made in reducing atherosclerosis-associated mortality in western countries, this pathology remains a global health issue. Over decades, a plaque grows and narrows the arteries’ lumen, leading to clinical manifestations such as myocardial infarction, stroke, and peripheral artery disease. Atherosclerotic plaques build up inside the large and median arteries (aorta, carotids, femoral arteries) due to the deposition of fat, cholesterol, calcium, fibrotic tissue, cells, and cellular debris. The development of atherosclerosis involves the activation of various cell types (including endothelial cells, smooth muscle cells (SMC), lymphocytes, monocytes, and macrophages) in the intima of the arteries, which results in a local inflammatory response. An increase in circulating low density lipoprotein (LDL)-cholesterol levels, and the subsequent accumulation of oxidized LDL (Ox-LDL) in the subendothelial space, triggers the recruitment and retention of monocytes and lymphocytes in the arterial wall. In the intima, monocytes differentiate into macrophages, which then scavenge lipoproteins, particularly Ox-LDL, and accumulate lipids, mainly cholesterol (1). One of the most important functions of macrophages in the context of atherosclerosis is the handling of cholesterol. The maintenance of macrophage cholesterol homeostasis is of critical importance because an imbalance between cholesterol influx and efflux leads to an excessive accumulation of cholesterol in macrophages and their transformation into foam cells (2, 3).

Macrophages express different scavenger receptors, including SR-A1, CD36, and lectin-like oxLDL receptor-1 (LOX-1), allowing internalization of lipoproteins, particularly Ox-LDL. They also express cholesterol transporters, including ATP-binding cassette transporters (ABCA1, ABCG1), and SR-BI, involved in the reverse cholesterol transport allowing elimination of excess unesterified cholesterol. Esterified cholesterol contained in Ox-LDL is internalized by phagocytosis and transported to late endosomes/lysosomes, where lysosomal acid lipase (LAL) hydrolyzes it and releases unesterified cholesterol, that is subsequently processed by acetyl-CoA acetyltransferase (ACAT1), in the endoplasmic reticulum to generate cholesteryl esters that accumulate in the cytoplasm leading to the formation of foam cells. On the other hand, newly synthesized cholesteryl esters can be hydrolyzed by neutral cholesterol ester hydrolase (NCEH), to generate free cholesterol that can leave the cells through the cholesterol transporters. Furthermore, other cell types, particularly SMC, can also become foam cells (4). Moreover, large amounts of intracellular unesterified cholesterol accumulation in the endoplasmic reticulum (ER) alters the function of integral ER membrane proteins, induces the ER stress signal transduction pathway, leading to an apoptotic response in the macrophages. *In vivo* evidence suggests that this event may promote plaque destabilization in advanced atherosclerotic lesions (5). Additionally, these macrophage-derived foam cells secrete inflammatory molecules and factors that further promote lipoprotein retention, affect SMC phenotype, proliferation, and migration from the media to the intima and sustain inflammation (2). Infiltrated macrophages also secrete matrix metalloproteases (MMPs) and cysteine endoproteases leading to matrix degradation and plaque rupture (6).

Another important factor regulating plaque stability, in addition to the accumulation of foam cells, SMC differentiation/proliferation/migration, and extracellular matrix degradation, is vascular calcification (VC), corresponding to the deposition of calcium crystals in the vascular wall (7). VC is associated with increased overall mortality and cardiovascular events (8). An important initiator of VC is cell death and, in this review, that is part of the article collection “Differential Activation of Cell Death Pathways in Macrophages as a Result of Adaptation to Divergent Microenvironment”, we focus on the role of macrophage cell death in atherosclerosis, and its potential role in VC.

## 2 Atherosclerosis and macrophage death

**Figure 1:**
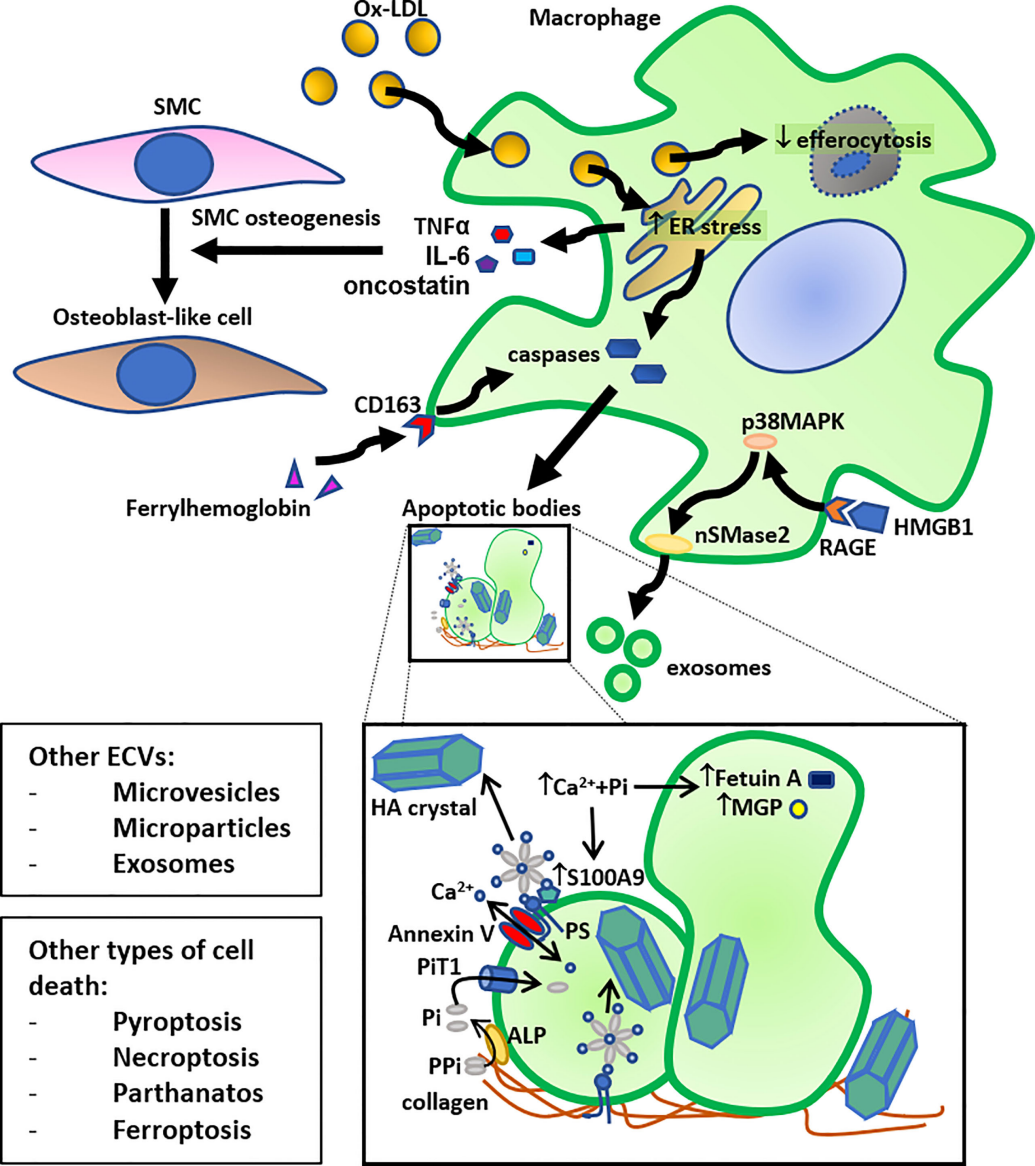
Schematic summary of the role of macrophage death in calcification. Macrophage cholesterol loading by oxidized LDL (Ox-LDL) (foam cell formation) leads to ER stress, followed by activation of caspases leading to apoptosis and release of apoptotic bodies. Another pathway that can induce macrophage apoptosis, followed by release of apoptotic bodies, is the ferrylhemoglobin internalization by CD163. Furthermore, high mobility group box 1 (HMGB1), released by dying cells, can induce exosome secretion by macrophages through activation of neutral sphingomyelinase-2 (nSMase2) by the receptor for advanced glycation end products (RAGE)/p38 mitogen-activated protein kinase (p38MAPK) pathway. The resulting apoptotic bodies and exosomes, or other secreted extra-cellular vesicles (ECVs), such as microvesicles and microparticles, bind to collagen in the extracellular matrix and act as calcium binding sites for hydroxyapatite (HA) crystals. More specifically, an annexin V - phosphatidylserine (PS) - S100A9/MRP14 membrane complex on the surface of ECVs acts as a nucleation site for HA crystal formation. More specifically, the anionic PS serves as a binding site for cationic Ca^2+^. HA crystals can also bind to collagen directly through electrostatic interactions; cationic moieties on collagen interact with negatively charged groups on HA crystal surface. Inorganic phosphate (Pi), derived from pyrophosphate (PPi) through alkaline phosphatase (ALP), can be transported into the ECV by the Pi transporter 1 (PiT1), and calcium can be transported by Annexin V. This accumulation of calcium and Pi also leads to HA crystal formation inside the ECV. An increase in the presence of calcium and inorganic phosphate (Pi) decreases the content of calcification inhibitors, such as Fetuin A and MGP, in these ECVs, and increases the presence of S100A9 on their surface, leading to further stimulation of HA crystal formation. Together, these mechanisms allow macrophages to contribute directly to VC. Macrophage foam cells also have reduced efferocytosis, and these lipid-loaded stressed macrophages secrete osteogenic factors, such as TNF-α, IL-6, and oncostatin, that stimulate trans-differentiation of smooth muscle cells (SMC) to osteoblast-like cells, thereby also indirectly contributing to VC. Lastly, other forms of cell death, such as pyroptosis, necroptosis, parthanatos, and ferroptosis can also lead to similar induction of calcification.

Apoptosis can occur in different cell types involved in atherosclerosis, such as SMC, endothelial cells, T lymphocytes and macrophages. Apoptotic macrophages account for more than 40% of dead cells (9). Apoptotic cell death progresses through several stages, initiating with nuclear chromatin condensation, followed by membrane blebbing, leading to disintegration of the cellular content into distinct membrane enclosed vesicles called apoptotic bodies (10). In addition, atherosclerotic plaque macrophages can undergo other types of cell death then apoptosis and necrosis. Indeed, while apoptosis remains the predominant form of macrophage death in atherosclerosis, less-well characterized types of regulated necrosis, such as pyroptosis, necroptosis, parthanatos and ferroptosis have been reported (11, 12) (**Figure 1**). Pyroptosis occurring in response to bacterial infection, is accompanied by NLRP3 inflammasome activation and by maturation of pro-inflammatory cytokines IL-1β and IL-18 (13, 14). Necroptosis is a form of cell death activated by death receptors, such as TNFR1, IFNR and TLR3/4 (15). The presence of lipopolysaccharide (LPS) from Escherichia Coli and Toll-like receptor 4 (TLR4) has been studied in specimens from carotids and controls matched patients. Immunochemistry analysis shown positive signals for LPS and TLR4 coincidentally with positivity for CD68 in the atherosclerotic plaque of carotids and the positivity for LPS and TLR4 was greater in the area with activated macrophages. These data provide the first evidence that LPS from Escherichia Coli localizes in human plaque and may contribute to atherosclerotic damage via TLR4-mediated oxidative stress (16). However, this link between infection and atherosclerosis is controversial and another mechanism was proposed where oxLDL can induce sterile inflammation through CD36-TLR4-TLR6 activation (17). Parthanatos is a mitochondrial-dependent cell death, triggered by DNA damaging stimuli, such as peroxynitrite or reactive oxygen species (ROS)-dependent activation of poly ADP-ribose polymerase (PARP)1 (18). Finally, ferropoptosis is a newly identified type of cell death caused by accumulation of iron-dependent lipid peroxides (19). The clearance of apoptotic cells is mostly mediated by macrophages, which recognize and internalize dead cells (endothelial cells, SMC, lymphocytes, senescent erythrocytes, neutrophils.) in a process termed efferocytosis (20–23).

In early lesions, phagocytes readily clear apoptotic cells, avoiding further progression of atherosclerosis, thus rendering plaques small and stable. During atherosclerotic plaque progression, ROS are abundantly produced by macrophages, leading to the formation of Ox-LDL that compete with apoptotic cells, interact with receptors of phagocytes thus leading to a reduced efferocytic capacity (24). This is one of the mechanisms by which Ox-LDL reduced efferocytosis. Moreover, it has been reported that the 7 keto-cholesterol (7KC) contained in Ox-LDL, and accumulating in macrophages and foam cells, impairs macrophage efferocytosis by a mechanism involving catabolism of phosphatidylinositol 4.5-biphosphate (PtdIns (4,5)P_2_) impairing actin depolymerization required for the completion of phagocytosis (25). Indeed,7KC inhibits both Fcγ receptor mediated and efferocytotic pathway by impairing phospholipase Cγ (PLCγ) thus preventing hydrolysis of PtdIns (4,5)P_2._

Additionally, transformation of macrophages into foam cells also diminishes their capacity to clear apoptotic debris (26). Therefore, in chronic, advanced lesions, the combination of high levels of macrophage apoptosis and defective efferocytosis causes accumulation of dead pro-inflammatory macrophages that can undergo post-apoptotic necrosis, leading to the formation of a necrotic core which contributes to plaque inflammation, instability and rupture (21, 27). Moreover, in atherosclerotic lesions, macrophages are exposed to different environmental stimuli (such as modified lipids, cytokines, and senescent erythrocytes) that dictate their functional phenotype. Indeed, several sub-classes of macrophages have been described in atherosclerosis, defined by the expression of specific surface markers, production of specific factors and biological functions (28). In humans, pro-inflammatory macrophages have been detected in the necrotic core of the plaque, differentiated in the presence of Ox-LDL, cholesterol crystals and pro-inflammatory cytokines, and producing pro-inflammatory factors. Anti-inflammatory, reparative macrophages have also been identified in human plaques. Their differentiation is drawn by different stimuli (IL-4, IL-13, oxidized phospholipids, CXCL4). They have the property to produce anti-inflammatory factors and are generally resistant to lipid accumulation and foam cell formation (28). Foam cells have been described as being less inflammatory than non-foamy cells (29). Moreover, anti-inflammatory macrophages located in areas of neo-vascularization or outside the lipid core are very effective in efferocytosis and phagocytosis since they highly express opsonins and receptors (30, 31). Notably, anti-inflammatory macrophages express high levels of C1qa, C1qb, C1qc, GAS-6 and thrombospondin-1. Since C1q deficiency leads to defective clearance of apoptotic cells (32), the high levels of several opsonins expressed in anti-inflammatory macrophages provide the molecular basis for their high phagocytotic capacity and efferocytosis of apoptotic pro-inflammatory macrophages and senescent erythrocytes, thus contributing to inflammation resolution (30, 32). Interestingly, pro-inflammatory macrophages are abundant in unstable human atherosclerotic plaques, whereas more stable plaques are enriched in anti-inflammatory macrophages (33).

## 3 Vascular calcification in atherosclerosis

VC can be located in the media (34), especially in patients with type 2 diabetes (35) and chronic kidney disease (36), and originates from the osteochondrogenic differentiation of SMC which share a common origin with osteoblasts, cells responsible for bone mineralization (37). Medial calcification is characterized by diffuse mineral deposition throughout the vascular tree and occurs independently of atherosclerosis. When media calcification, also called Mönckeberg’s medial sclerosis (38), affects the aorta and large arteries (such as the iliac arteries and the arteries of the lower limbs), it decreases their elasticity and causes hemodynamic alterations (39), leading to hypertension, aortic stenosis and left ventricular hypertrophy (40). Intimal VC, associated with inflammation and oxidative stress, as well as the development of atherosclerotic plaques and occlusive lesions (41), is more frequent at the coronary and carotid level, and is largely influenced by traditional cardiovascular risk factors (41).

As mentioned above, diabetes plays a major role in VC in the media and in Mönckeberg’s disease (35). In addition, diabetes also contributes to the development of atherosclerosis since early stages, and is associated with atherosclerotic plaque calcification, which occurs in the advanced stages of atherosclerosis (42).

In the general population, VC has a high heritable component, suggesting that in certain cases there is a genetic factor contributing to the disease (43). However, there are few monogenic disorders presenting with early-onset cardiovascular disease that can be classified into disorders caused by an altered purine and phosphate/pyrophosphate metabolism (calcification of joints and arteries, generalized arterial calcification of infancy, Hutchinson-Gilford progeria syndrome, idiopathic basal ganglia calcification, pseudoxanthoma elasticum, interferonopathies (Singleton-Merten Syndrome), and Gaucher disease) (44).

Two types of calcifications have been reported in atherosclerotic plaques, based on their size. Initially, microcalcifications occur (45, 46) with a size lesser than 50 μm, indetectable by conventional imaging methods (47). They are identified in unstable plaques (48), favoring their rupture (49) and are associated with the presence of macrophages with pro-inflammatory activity (50). Secondarily, these microcalcifications coalesce to form macrocalcifications, with a size greater than 50 μm, present in stable plaques rather associated with anti-inflammatory macrophages (51). The calcium crystals present in atherosclerotic plaques, can be of different nature, including crystals of calcium phosphate (hydroxyapatite) which are predominant, calcium carbonate, calcium oxalate, dicalcium phosphate dihydrate and octacalcium phosphate (52). VC is a dynamic regulated process sharing many features with bone formation, implicating both stimulating and inhibitory factors, mediated by osteoblast-like and osteoclast-like cells, respectively, and involving particularly SMC and macrophages. Osteoclasts originate from hematopoietic lineage cells derived from bone marrow myeloid precursors or circulating monocytes (53). The presence of osteoclast-like cells in the vascular wall is rather limited, possibly because factors that inhibit/modulate the differentiation of monocyte/macrophages into osteoclast-like cells are present (54). In atherosclerotic lesions, macrophages that express both the MCSF and RANKL receptors (55) can be activated by their ligands produced by endothelial cells, SMC, and monocyte/macrophages themselves, and can thus potentially differentiate into osteoclast-like cells. Moreover, the presence of macrophages displaying an anti-inflammatory phenotype has been reported in areas surrounding calcium deposits in human atherosclerotic plaques (56). However, these anti-inflammatory macrophages express carbonic anhydrase type II (degrading the inorganic part of mineralized extra-cellular matrix), but relatively low levels of cathepsin K, involved in the degradation of the organic part of extracellular matrix, allowing them to be phenotypically defective to resorb calcification (56). Concerning the extra-cellular matrix mineralization, in the presence of Ox-LDL, macrophages produce TNFα, that enhances SMC osteoblastic-like trans-differentiation, alkaline phosphatase (ALP) expression and matrix mineralization (57). Moreover, macrophages express several bone-related proteins, such as matrix Gla protein (MGP), ALP, bone sialoprotein and osteopontin (58). In response to various pro-osteogenic signals such as high glucose, inflammation, and oxidative stress, SMC undergo an osteoblast-like trans-differentiation by acquiring an osteoblastic phenotype and become able to deposit calcium and other minerals within the plaque (59) a process that requires the expression of osteogenic transcription factors such as RUNX2 (60), as well as enzymes such as ALP.

### 3.1 Vascular calcification and apoptosis

Cell apoptosis from SMC and/or macrophages, is considered as one of the main drivers of calcification in humans. Indeed, post confluent human SMC spontaneously form nodules in cell culture and induce calcification, as detected by von Kossa’s method, Alizarin red S staining, and electron microscopy (61). Apoptosis induction of these cells with anti-Fas IgM and cycloheximide for 24 hours, demonstrated by Tunel labeling and phosphatidyl serine exposure, increased nodule calcification (61). Moreover, apoptosis inhibition with the cell permeable caspase inhibitor ZVAD.fmk decreases calcification, as assessed by alizarin red staining (61). Caspase inhibition can also lead to a reduced release of apoptotic bodies from cells (61). Interestingly, DNA damage characteristics were observed after 7 days of culture, while the calcification occurs after 28 days (61), thus suggesting that apoptosis precedes calcium crystal formation. Moreover, SMC-derived apoptotic bodies initiate calcification in a similar way as already reported for chondrocytes in cartilage (62). These apoptotic bodies accumulate calcium and need an integral membrane; phosphatidyl serine exposure by apoptotic bodies generates potential calcium binding sites suitable for hydroxyapatite deposition (61). Moreover SMC-derived matrix vesicles are also involved in the initiation of VC (63). These vesicles contain apoptotic proteins, such as BAX, suggesting that they may be remnants of apoptotic cells (64). Some studies have suggested that apoptotic bodies in atherosclerotic plaques are like matrix vesicles and may initiate calcification (65, 66). However, much controversy exists in the field concerning the classification and nomenclature used to classify different extra-cellular vesicles (ECVs), depending on the method of purification, the surface marker used, the morphology, selected size, etc … It’s actually admitted that the terms ECVs includes exosomes, microvesicles, microparticles, and apoptotic bodies (67). These ECVs, that can physiologically derive from macrophages, SMC, and endothelial cells, are microscopic phospholipid bilayer-enriched particles, of round or ovoid shape, covering size from approximatively 30 nm to 5 µm (68, 69). They are often associated with extracellular matrix components, particularly collagen, and displaying evidence of hydroxyapatite crystals on the inner membrane within the lumen, and on the outer membrane of the vesicle. Under physiological conditions, these ECVs are enriched in calcification inhibitors, such as fetuin A or MGP (70). When exposed to an excess of calcium and phosphate, the concentration of these inhibitors is reduced and ECVs become enriched in annexin V-and phosphatidylserine that allow calcification by inducing collagen binding (71) (**Figure 1**). Conditioned media collected from human coronary SMC cultured for 14 days in calcifying conditions (medium containing dexamethasone, L-ascorbic acid and β-glycerophosphate) was used to isolate ECVs by ultracentrifugation. The latter have been fluorescently labelled and added to collagen hydrogels, resulting in a progressive formation of ECV aggregates, between collagen fibers. In this way, calcium and phosphate nucleate to form hydroxyapatite leading to the appearance of dense calcifying structure like those observed in human calcified plaques (72). Thus, collagen serves as a scaffold directing ECVs aggregation and microcalcification formation. Calcifying ECVs have been reported in medial and intimal calcification (73), as well as in calcified aortic valves (74) and appear similar to the ECVs involved in physiological bone mineralization (75).

### 3.2 Role of macrophage death in the initiation and progression of calcification

While the role of SMC apoptosis in initiation of calcium deposits is well documented and established in the literature (61), those of macrophages is still in its infancy, particularly because the direct role of macrophages in VC is quite novel. Indeed, while it is accepted that macrophages can modulate SMC mineralization by indirect paracrine mechanism, through the secretion of pro-osteogenic cytokines and factors (57), their direct role in VC, as cellular actors able to mineralize the extra-cellular matrix is more recent. Thus, further studies are necessary to understand the role of macrophage apoptosis in VC.

However, deep histological analysis of human samples, revealed that apoptotic macrophages, more often apoptotic foam cells, are present in areas of calcium deposits as microcalcifications (76). These calcifications form into the lipid core in early fibroatheroma where macrophages infiltrate the plaque, undergo apoptosis and release matrix vesicles (77). Macrophage apoptosis may result in a different morphology of calcium deposits, compared to apoptotic SMC. Indeed, while SMC apoptosis led to fine microcalcifications, those derived from apoptotic macrophages are characterized by large, punctate and blocky appearance (76). These calcifications, generally observed in the deeper areas of the necrotic core, close to the internal elastic lamina (78), then coalesce to form larger calcification areas, called “fragmented” calcification, in a process involving both the necrotic core as well as the surrounding extra-cellular matrix (77). One of the more accepted hypothesis is that SMC apoptosis is the driving force for microcalcification (77), followed by macrophage infiltration into the lipid core where they also undergo cell death and calcification. Cell death provides phospholipid rich debris that serve as nucleation site of hydroxyapatite, a process that starts within lipid pools and progresses with inflammation and further cell death, leading to the development of necrotic core (78). Recently, an oxidative form of hemoglobin (ferrylhemoglobin) has been detected in complicated human atherosclerotic lesions. This ferrylhemoglobin, which is internalized by macrophages through the CD163 pathways, affected macrophage polarization, induced apoptosis as well as calcification (79) (**Figure 1**). Interestingly, while intraplaque hemorrhage has been associated with calcification and may facilitate its progression (80), a recent study demonstrated a mechanism by which CD163+ macrophages can inhibit VC through NF-κB–induced enhanced production of the anti-calcific glycosaminoglycan, hyaluronan (HA) (81).

Mechanistical data supporting the direct role of macrophage apoptosis/death in the process of VC are rare. Moreover, macrophages are an important source of ECVs that act as hydroxyapatite nucleation initiators. Indeed, microcalcifications in vulnerable plaques contain more macrophages than SMC in stable plaques (45, 73). When stimulated by phosphate and calcium, *in vitro* RAW 264.7 murine macrophages increase the number of released pro-calcifying EVCs, expressing exosomal markers (CD9 and TSG101) and being enriched in S100A9, also known as migration inhibitory factor-related protein 14 (MRP-14) and annexin V (**Figure 1**). Silencing S100A9 *in vitro* as well as deletion in S100A9-/- deficient mice reduced EVC calcification, whereas stimulation with S100A9 increased their calcification potential. The calcium/phosphate induction of phosphatidylserine-annexin V-S100A9 membrane complex in these EVCs facilitates the hydroxyapatite nucleation in the cell membrane (73). LPS-stimulation of RAW 264.7 murine macrophages decreased the number of ECVs, but these were able to induce inflammation and oxidative stress in SMC, resulting, under calcifying conditions, in an increased expression of osteogenic markers accompanied by an induced calcification and a decrease in contractile marker expression (82). These observations were specific to the LPS-induced ECVs, since LPS alone did not modulate calcification.

Moreover, the role of Transient Receptor Potential Canonical 3 (TRPC3), a non-selective calcium-permeable channel and obligatory signaling component of the ER stress-induced apoptosis (83) has been evaluated. Transfer of bone marrow with deficiency of the TRPC3into LDL-R KO recipient mice, led to a decrease in necrosis and number of apoptotic pro-inflammatory macrophages (84), compared to control mice, suggesting a more stable plaque phenotype. Interestingly, these mice were also characterized by a decreased calcification of the aortic roots, an effect accompanied by a significant reduction of the plaque expression of osteogenic markers, such as BMP-2, ALP and RUNX2 (85, 86). Since apoptotic bodies are important centers for nucleation, it can by hypothesized that the reduced calcification observed in this model is in part due to the reduced number of apoptotic macrophages present in the plaque. These data suggested that macrophage ER stress induced apoptosis can be targeted to control calcification. Disruption of ER homeostasis causes accumulation of unfolded and misfolded proteins in the ER lumen, leading to activation of ER stress signaling. ER stress signaling is composed of three signaling axes that are initiated by inositol-requiring protein-1 (IRE1), double-stranded RNA-dependent protein kinase-like ER kinase (PERK) and activating transcription factor 6 (ATF6). During ER homeostasis, PERK, ATF6, and IRE1 combine with the molecular chaperone GRP78T to form a stable complex. Under ER stress, these proteins disaggregate and stimulate three important signaling pathways, resulting in the expression of apoptosis protein CHOP and the activation of Jun-N-terminal kinase (JNK) to promote apoptosis (87).

PARP1 has been reported to contribute to foam cell death, particularly parthanatos, an important determinant of plaque composition (88). Interestingly, specific PARP1 macrophage deletion in diabetic apolipoprotein E deficient mice, attenuates the formation of calcium nodules in atherosclerotic lesion and significantly decreases the total calcium content of the aorta (89). *In vitro* experiments indicated that PARP1 inhibition reverses the high glucose-induced calcification as well as RUNX2 expression, by a mechanism involving STAT1 pathway. These results suggest that PARP1 inhibition decreases the osteogenic like potential of macrophages. Moreover, PARP1 inhibition leads to a shift of macrophage polarization toward an anti-inflammatory phenotype (89). Indeed, different PARP1 inhibitors have demonstrated atheroprotective effects by decreasing PARP activation, inflammatory markers, macrophage recruitment, endothelial dysfunction, foam cell death thus promoting plaque stability (88).

Finally, High mobility group box protein 1 (HMGB1) associated with macrophage pyroptosis  (90), accumulated in areas of calcification (91) and has been identified as potential inducer of ECVs secretion by macrophages (92). HMGB1can induce exosome secretion by activating the neutral sphingomyelinase-2 (nSMase2) by the receptor for advanced glycation end products (RAGE)/p38 mitogen-activated protein kinase (p38MAPK) pathway. Indeed, failure in apoptotic bodies cleaning by macrophages allow calcium crystals to grow (93) and also result in the release of pro-inflammatory cytokines (such as TNFα, IL-6, and oncostatin M) able to modulate VC by inducing osteogenic gene expression in SMC (57, 94, 95). Interestingly, IL-1β released by macrophages during pyroptosis by a caspase-1 dependent mechanism, enhanced SMC osteogenic differentiation and subsequent calcification (96).

## 4 Conclusion

If we consider that in advanced atherosclerotic plaques the efferocytosis capacity of macrophages decreased, this will lead to a greater availability of apoptotic bodies and necrotic cells which could serve as a nucleation starter site for the formation of calcium crystals. Additionally, defective cleaning of apoptotic cell bodies by macrophages, derived from macrophages themselves or from SMC (97), will results in the release of inflammatory cytokines, also able to impact calcification (98). This may be a plausible explanation for the observation that calcification preferentially develops in advanced plaques.

Moreover, it has been reported that anti-inflammatory macrophages located around the calcium deposits in human plaques, are highly efferocytotic (30). However, the capacity of these macrophages to directly participate to VC is under investigated, even though they display a defective osteoclast-like phenotype but appear to be able to participate to extracellular matrix mineralization (56) and we can hypothesize that their efferocytosis effectiveness could be also modified by the presence of calcium deposits. It would thus be necessary to better study the impact of osteoclast defective phenotype and enhanced efferocytosis of these macrophages in terms of calcification. We can thus hypothesize that macrophages can be crucial in the control of calcium deposition in atherosclerosis, since they are cells highly specialized in efferocytosis, thus reducing the number of environmental apoptotic cells. Considering that inhibition of apoptosis results in inhibition of calcification (61), we can speculate that targeting macrophage efferocytosis may be a promise way to reduce VC as well as general plaque progression.

## Author contributions

Bibliography search, writing and editing: JN, CG and GC. All authors have read and agreed to the published version of the manuscript.
